# SARS-CoV-2 Receptors are Expressed on Human Platelets and the Effect of Aspirin on Clinical Outcomes in COVID-19 Patients

**DOI:** 10.21203/rs.3.rs-119031/v1

**Published:** 2020-12-23

**Authors:** Aditya Sahai, Rohan Bhandari, Milka Koupenova, Jane E. Freedman, Matthew Godwin, Thomas McIntyre, Mina K. Chung, Jean-Pierre Iskandar, Hayaan Kamran, Essa Hariri, Anu Aggarwal, Ankur Kalra, John R. Bartholomew, Keith R. McCrae, Ayman Elbadawi, Lars G. Svensson, Samir Kapadia, Scott J. Cameron

**Affiliations:** 1 Heart Vascular and Thoracic Institute, Cleveland Clinic, Cleveland, OH; 2 Department of Cardiovascular and Metabolic Sciences, Cleveland Clinic, Cleveland, OH; 3 Department of Medicine, University of Massachusetts Medical School, Worcester, MA.; 4 Case Western Reserve University Cleveland Clinic Lerner College of Medicine, Cleveland, OH; 5 Department of Medicine, Cleveland Clinic, Cleveland, OH; 6 Taussig Cancer Institute, Cleveland Clinic, Cleveland, OH; 7 Division of Cardiovascular Medicine, University of Texas Medical Branch, Galveston, TX

**Keywords:** Platelets, SARS-CoV-2, COVID-19, Thrombosis, ACE2, TMPRSS2

## Abstract

Coronavirus disease-2019 (COVID-19) caused by SARS-CoV-2 is an ongoing viral pandemic marked by increased risk of thrombotic events. However, the role of platelets in the elevated observed thrombotic risk in COVID-19 and utility of anti-platelet agents in attenuating thrombosis is unknown. We aimed to determine if human platelets express the known SARS-CoV-2 receptor-protease axis on their cell surface and assess whether the anti-platelet effect of aspirin may mitigate risk of myocardial infarction (MI), cerebrovascular accident (CVA), and venous thromboembolism (VTE) in COVID-19. Expression of ACE2 and TMPRSS2 on human platelets were detected by immunoblotting and confirmed by confocal microscopy. We evaluated 22,072 symptomatic patients tested for COVID-19. Propensity-matched analyses were performed to determine if treatment with aspirin or non-steroidal anti-inflammatory drugs (NSAIDs) affected thrombotic outcomes in COVID-19. Neither aspirin nor NSAIDs affected mortality in COVID-19. However, both aspirin and NSAID therapies were associated with increased risk of the combined thrombotic endpoint of (MI), (CVA), and (VTE). Thus, while platelets clearly express ACE2-TMPRSS2 receptor-protease axis for SARS-CoV-2 infection, aspirin does not prevent thrombosis and death in COVID-19. The mechanisms of thrombosis in COVID-19, therefore, appears distinct and the role of platelets as direct mediators of SARS-CoV-2-mediated thrombosis warrants further investigation.

## Introduction

COVID-19 is caused by the severe acute respiratory syndrome coronavirus-2 (SARS-CoV-2) and curiously displays a propensity for thrombosis in multiple vascular beds. COVID-19-related thrombosis may contribute to severe organ injury and death. The incidence of thrombotic events was as high as 31% in one cohort ^1^. Clinical and autopsy studies of COVID-19 patients suggest an increased risk of microthrombi, venous thromboembolism (VTE), and ischemic stroke^2,3^. Activated platelets are circulating mediators of thrombosis and, therefore, may serve as a logical therapeutic target in COVID-19. Two registered clinical trials (NCT04363840 and NCT04365309) will prospectively evaluate patient outcomes following low dose aspirin in the context of SARS-CoV-2 infection.

SARS-CoV-2 utilizes an spike glycoprotein to bind to the host transmembrane angiotensin-converting enzyme 2 (ACE2) and is then cleaved by the serine protease TMPRSS2 to coordinate entry into the host cell^4,5^. Therefore, co-expression of ACE2 and TMPRSS2 may be important for host cell entry and infectivity of SARS-CoV-2. Importantly, human tissue distribution of ACE2 and TMPRSS2 mirrors organ system involvement in COVID-19 and includes the lungs^6-11^, vascular endothelium^9-12^, heart^11,13,14^, kidneys^8,10,13^, liver^8,10^, digestive tract^8,10,11,15^, nasal epithelium^7,10,11^ and central nervous system^10,14^. Single-stranded RNA (ssRNA) viruses, including influenza, are engulfed by platelets and may contribute to immuno-thrombosis indirectly through developing neutrophil extracellular traps (NETs) by engaging the platelet toll-like receptor 7 (TLR7)^16^. SARS-CoV-2, another SSRNA virus, utilizes platelets to modulate immunologic responses including the development of neutrophil extracellular traps (NETs) that are emerging as pro-thrombotic responses in patients with COVID-19^17^. Further, elevation of soluble P-selectin and sCD40L in blood from patients with COVID-19 compared to controls provides indirect evidence of platelet activation in COVID-19 coagulopathy^18^. SARS-CoV-2 is a ssRNA virus, and therefore may directly augment platelet activation causing myocardial infarction (MI), stroke, and VTE.

A recent report demonstrated that COVID-19 patients have a divergent platelet transcriptome from healthy individuals, and aspirin suppresses COVID-19 platelet activation *in vitro*^19^. The platelet surface receptor for SARS-CoV-2 was not clarified in this study, while a similar investigation by another group identified mRNA for SARS-CoV-2 in human platelets^20^. Thus, our goal was to determine if platelets express known SARS-CoV-2 receptor proteins and, as with influenza previously, contribute to thrombotic events in patients. In the absence of clinical trial data, we sought to evaluate the potential benefit in mitigating thrombotic responses *in vivo* with use of aspirin or other NSAID antiplatelet therapies by propensity matching patients using real-world data.

## Methods

### Platelet Isolation

Healthy volunteers without any known medical history or on antiplatelet therapy donated blood specimens in accordance with and approved by the Cleveland Clinic Foundation Institutional Review Board (IRB) approval. For each subject, venous blood was drawn by a medical professional into citrate plasma tubes, then centrifuged in a tabletop centrifuge at 1100 RPM for 15 minutes. The platelet rich plasma (PRP), collected well above the buffy coat, was decanted and the platelets were centrifuged at 2600 RPM for an additional 5 minutes. These washed platelets were then used in immunoblotting and fluorescence-activated cell sorting (FACS) analyses.

### Immunoblotting

Washed platelets from healthy subjects or patients with coronary artery disease (CAD) enrolled at the Cleveland Clinic main campus in Ohio were isolated and proteins separated by SDS-PAGE as we have previously documented ^21,22^ and in accordance with IRB protocols (#19-1451 for patients and #20-413 for healthy volunteers). We utilized human brain lysate, human placenta, and engineered human heart tissue as positive controls for TMPRSS2 and ACE2. Human brain lysate is commercially available (Novus #NB820-59177). Human placenta lysate was prepared as follows: placental villous tissue was collected immediately upon uncomplicated, full-term (37–42 weeks’ gestation), elective C-section deliveries at MetroHealth Hospital in Cleveland, Ohio and approved by the Cleveland Clinic and MetroHealth IRB (#16–1311 and #16–00335, respectively). This tissue was normally discarded placentas with intact fetal membranes, and following inclusion in the study no protected health information, identifiers, or clinical data were collected. A waiver of consent was approved by the Cleveland Clinic Foundation IRB as the placentas were collected anonymously. Engineered human heart tissue was obtained as follows: human-induced pluripotent stem cells (generated by the California Institute of Regenerative Medicine) were differentiated into beating ventricular-like cardiomyocytes (iCMs) and grown in a monolayer. To enhance maturation, iCMs were subsequently grown as engineered heart tissues as we have previously described^23^. Immunoblotting was conducted using anti-TMPRSS2 (abcam #92323), anti-ACE2 (Abcam #15348), anti-tubulin (CST #3873S), and anti-GAPDH (CST #5174) antibodies. The mean ratio of TMPRSS2 or ACE2 to loading control ± SEM is documented, unless stated otherwise. Primary antibody was used as in a 1:10000 titer overnight at 4°C in 3% bovine serum albumin/Tris-buffered saline-Tween 20. Secondary antibody (GE Healthcare, Buckinghamshire, UK) was used in a 1:2000 titer in 5% milk/Tris-buffered saline-Tween for 1 hour at room temperature. Final autoradiographic films (Bioblot BXR, Laboratory Product Sales, Rochester, NY) were quantified by densitometry using ImageJ software (National Institutes of Health). All experiments were performed in accordance with relevant guidelines and regulations.

### Confocal Microscopy

Venous blood drawn into and separated as citrated plasma was lysed and fixed with BD FACS lysing solution (BD Biosciences, NJ, USA, cat# 349202) for 10 mins. The platelet pellet was washed with 1X PBS, centrifuged at 1500g for 7 mins, resuspended in HEPES-buffered Tyrode solution supplemented with 2% FBS and then stained for 1 hour with the following: CD41 to confirm platelets (ThermoFisher eBio cat #11-0419-42), ACE2 antibody (Novus cat#NBP2-72117AF647), TMPRSS2 antibody (SantaCruz cat#sc-515727 AF488) and DAPI to eliminate any DNA components. Mounted slides were resolved by fluorescent microscopy using a Scanning Disk Nikon A1 confocal microscope with 100x objective lens. All experiments were performed in accordance with relevant guidelines and regulations.

### Study Design

Quality-assured clinical data from ambulatory and hospitalized Cleveland Clinic patients treated in Northeast Ohio and South Florida was used to appraise data on 22,072 symptomatic patients evaluated for COVID-19 with the goal of determining whether current aspirin use protects patients from death and/or the secondary composite outcome of MI, thrombotic stroke, and/or VTE. Positive testing for a SARS-CoV-2 amplicon by nasopharyngeal RT-PCR was used to determine infection status. The electronic medical record and hospital Medication Administration Record (MAR) was used to confirm new or ongoing administration of 81 mg aspirin or other NSAIDs for both outpatients and inpatients.

### Statistical Analysis

Categorical factors are summarized using frequencies and percentages, while continuous factors are described using median and ranges. Initial descriptive analyses were performed. Comparisons were made between those with known death status and those with missing death information to identify if any differences exist in these cohorts. Then among those with known death status, differences in COVID positive and COVID negative patients were assessed. Finally, after stratifying by COVID status, comparisons of those with and without aspirin use were performed. For all tables, continuous measures were compared using nonparametric Wilcoxon rank sum tests, while categorical factors were compared using Pearson chi-square tests or Fisher exact tests, for rare events.

Given the differences across many covariates, propensity score matching was performed to account for differences between those with and without aspirin use. This approach used two steps. First, multiple imputation was performed on all demographic and covariate measures within COVID status stratified datasets, using fully conditional specification methods. Ten imputed datasets were created. Then propensity score models were fit for each dataset, with aspirin use as the response and all other measures as predictors. Predicted probability of aspirin use from each model was calculated, and these probabilities were averaged across models for each patient. Greedy matching was then performed using a caliper of 0.2 standard deviations of the logit to create matched datasets for both COVID positive and negative patients. A small number of aspirin users could not be matched well and were excluded from the matched analysis. Comparisons of outcomes were performed using mixed effect logistic regression models to account for the matching process. Overlap weighting propensity score analyses were also performed^24^ which data with the same conclusions. This analysis was repeated using NSAID groups. For significant effects, E-values^25^ that represent the magnitude of the association between an unobserved covariate and both the medication group and outcome necessary to make the result non-significant was also calculated. Analyses were performed using SAS software (version 9.4; Cary, NC). A significance level of 0.05 was assumed for all tests.

## Results

**Figure 1.**
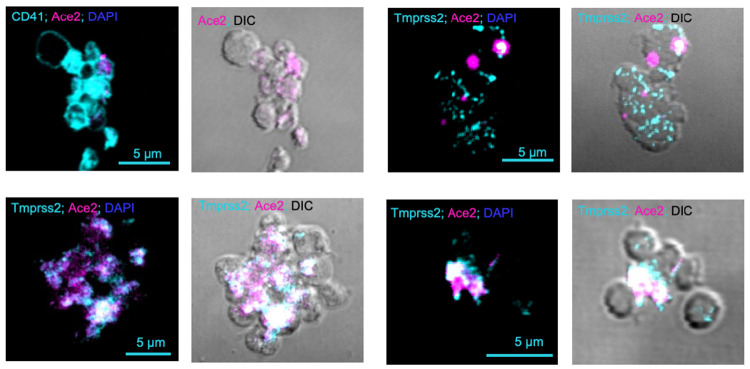
Expression of ACE2 and TMPRSS2 in Platelets by Confocal Microscopy. Platelets isolated from venous blood of healthy individuals was stained for 1h with the following antibodies: CD41 (platelet-specific marker), ACE2, TMPRSS2, and DAPI to eliminate any DNA components. Mounted slides were resolved by confocal fluorescent microscopy using a 100x objective lens. Images are representative of n=6 donors for ACE2 and n=3 for TMPRSS2. Each image represents a different donor. The scale bar is noted.

Expression of ACE2 (n=6) and TMPRSS2 (n=3) on the platelet surface was observed by confocal microscopy (Figure 1). Expression of TMPRSS2 in healthy subjects (mean age 40.1 ± 2.8 years, n=20) was also confirmed by immunoblotting at the expected molecular weight of ~50 KDa.

**Figure 2.**
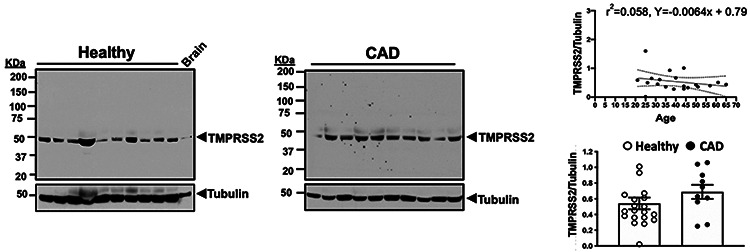

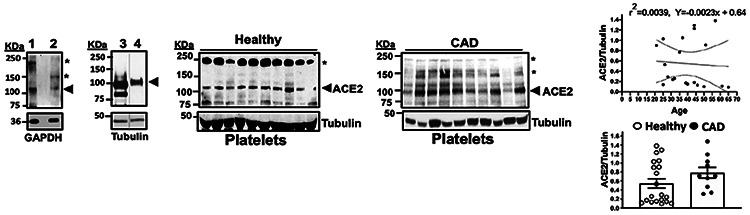
**A. Expression of TMPRSS2 in Platelets:** Washed platelets from healthy individuals (mean age 40.1 ± 2.8 years, n=20) were isolated and proteins separate by SDS-PAGE with molecular weight shown in KiloDaltons (KDa). Immunoblotting was conducted an using an anti-TMPRSS2 antibody or anti-tubulin immunoblotting as a loading control. The ratio of protein to loading control is expressed as a function of age and the correlation coefficient is noted (r ± 95% CI, P=0.30). Human brain lysate served as a positive control for TMPRSS2 migrating at the expected molecular weight (~50 KDa). Data shown are representative of 20 healthy individuals (10 male and 10 female) and 10 patients with coronary artery disease (CAD). The mean ratio of TMPRSS2/Tubulin ± SEM is noted, P=0.145 between healthy and CAD by Mann Whitney *U*). **B. Expression of ACE2 in Platelets:** Washed platelets from healthy individuals (mean age 40.1 ± 2.8 years, n=20) were isolated and proteins separate by SDS-PAGE with molecular weight shown in KiloDaltons (KDa). Lane 1 is human platelet lysate, lane 2 is human brain lysate, lane 3 is human placenta lysate, lane 4 is lysate from engineered human heart tissue. Immunoblotting was conducted using an using anti-ACE2 antibody. Anti-tubulin and anti-GAPDH are loading controls. ACE2 migrates at the expected molecular weight (~100 KDa) shown by an arrowhead with glycosylated forms indicated by *. The ratio of ACE2 protein to loading control is expressed as a function of age and the correlation coefficient is noted (r ± 95% CI, P=0.79). Data shown are representative of 20 healthy individuals (10 male and 10 female) and 10 patients with coronary artery disease (CAD). The mean ratio of ACE2/Tubulin ± SEM is noted, P=0.112 between healthy and CAD by Mann Whitney *U*).

Utilizing human brain as a positive control, TMPRSS2 expression was standardized to a loading control with no correlation between age and platelet TMPRSS2 expression (Figure 2A; r^2^=0.058, p=0.30). Since ACE2 exists as multiple glycosylated proteins of variable molecular weight^26-28^, human brain^29^, human placenta^30^, and engineered heart tissue^31^ were utilized as positive controls to confirm predominant migration at ~100 kDa as expected. Given that patients with confirmed CAD receive antiplatelet medications according to established guidelines, TMPRSS2 expression for healthy controls (n=20) was compared to patients with coronary artery disease (CAD, n=10) and, while numerically greater in CAD, was without a statistically significant difference (Figure 2A, p=0.15).

Similarly, expression of ACE2 in healthy subjects (n=20) was confirmed by immunoblotting. ACE2 expression standardized to tubulin did not correlate with age (Figure 2B; r^2^=0.0039, p=0.79).

Platelet ACE2 in healthy subjects (n=20) was compared to patients with CAD (n=10) and, again, while numerically higher in CAD, was without a statistical difference (Figure 2B, p=0.11). Further, we did not observe sex-specific differences in platelet expression of ACE2 or TMPRSS2 (20 men and 20 women in each group). Full size, uncropped immunoblots for ACE2, TMPRSS2, and loading controls are found in Supplemental Figure 1A-C.

**Figure 3.**
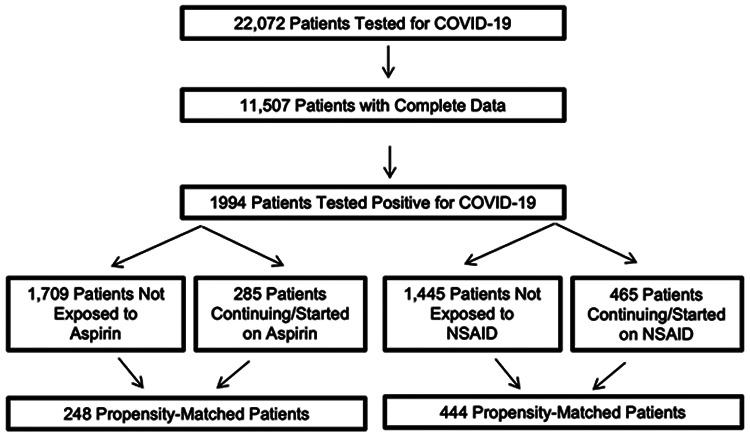
Patients Testing Positive for SARS-CoV-2 taking Aspirin or NSAIDs. Patients testing positive for a SARS-CoV-2 amplicon at two Cleveland Clinic hospitals were evaluated. Patients initiated with aspirin or NSAID therapy or continuing aspirin or NSAID if admitted to the hospital were included in this study. Clinical variables in each group where then re-evaluated following careful propensity matching

22,072 patients tested for COVID-19 at two Cleveland Clinic hospitals between March 13, 2020 to May 13, 2020 were evaluated. Within this cohort, 11,507 patients had complete clinical data and 1,994 tested positive for the SARS-CoV-2 amplicon by RT-PCR testing. Amongst these 1,994 patients, 1,709 were not exposed and 285 patients were exposed to aspirin. In an attempt to differentiate an anti-platelet drug effect with aspirin from a more general NSAID class effect, we propensity-matched patients 1,445 patients not exposed and 465 patients exposed to NSAID therapy (Figure 3).

**Figure 4.**
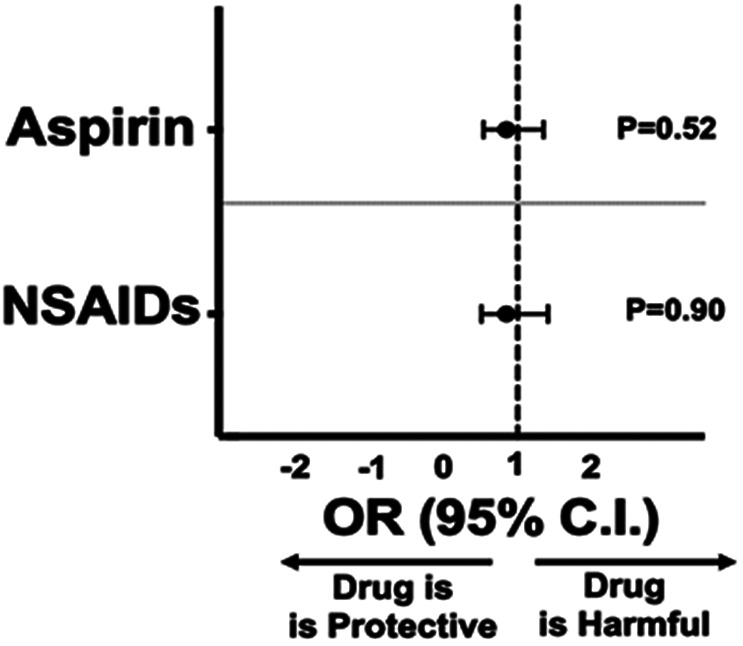
Mortality for Propensity-matched patients: Propensity-matched data for patients testing positive for COVD-19 and outcomes taking either 81 mg aspirin (n=248 in each group) or NSAIDs (n=444 in each group) at the time of diagnosis. Forest plot representation of data as Odds Ratio (OR) with 95% confidence interval (C.I.) for the primary endpoint of death

The 248 propensity-matched patients either treated with aspirin or not demonstrated no significant group differences in demographics or clinical covariates. Aspirin therapy did not alter mortality (13.3% vs 15.3%, p=0.53). The 444 propensity-matched patients either exposed or not to NSAIDs demonstrated no significant group differences in demographics or clinical covariates. NSAID therapy did not alter mortality (7.0% vs 7.2%, p=0.90). In propensity-matched patients treated with aspirin, the incidence of MI (2.0% vs 0.81%, p=0.27) and VTE (4.0% vs 1.6%, p=0.12) were not significantly different, but aspirin therapy was associated with an increased risk of thrombotic stroke (3.6% vs 0.40%, p=0.036). In propensity-matched patients treated with NSAIDs, the incidence of MI (0.68% vs 0.23%, p=0.34), VTE (2.0% vs 0.90%, p=0.17), and thrombotic stroke (1.1% vs 0.45%, p=0.27) was not affected. Using the composite thrombotic endpoint of MI, VTE, and thrombotic stroke, both aspirin (9.3% aspirin vs 2.8% no aspirin, p=0.005) and NSAID therapy (3.8% NSAIDs vs 1.6% no NSAIDs, p=0.046) were associated with signals for thrombosis (Supplemental Figure 1). Overall, there was no change in mortality in COVID-19 for patient treated with either aspirin (OR 0.52, 95% CI: 0.51-1.41; p=0.52) or NSAIDs (OR 0.97, 95% CI: 1.62; p=0.90) (Figure 4).

However, both aspirin and NSAID use in COVID-19 show signals for harm with increased thrombotic risk with aspirin (OR 3.52, 95% CI: 1.48-8.40; p=0.005) and NSAIDs (OR 2.49, 95% CI: 0.58-1.62; p=0.046) for the composite endpoint of MI, thrombotic stroke, and VTE (Supplemental Figure 2).

## Discussion

In this study, we make the observation that both ACE2 and TMPRSS2 proteins which bind and ligate SARS-CoV-2 are expressed in healthy human platelets. The expression of these receptors in platelets does not vary significantly with age and, while numerically higher, are not strikingly different in patients with CAD compared to healthy controls. The presence of known SARS-CoV-2 receptors on platelets suggests the possibility that SARS-CoV-2 may directly activate platelets and contribute to thrombosis or promote thrombosis indirectly by mediators secreted from platelets .

A recent investigation revealed platelet reactivity is enhanced in COVID-19 patients^20,32-34^ and appears to be suppressed by the presence of high dose aspirin *in vitro*^33^. In the absence of randomized controlled data for aspirin in patients with COVID-19, we conducted a propensity-matched analysis of patients showing aspirin has no mortality benefit in patients with COVID-19, and, in fact, displays a slightly increased signal for harm driven mostly by thrombotic stroke. Platelet reactivity data *in vitro* is often extrapolated to suggest a risk for harm, but it is important to acknowledge that the behavior of anti-platelet medications *in vivo* can be markedly different from *in vitro* studies. Our goal was to clarify this concern by using real-life data with both mortality and thrombotic end points.

The failure to show a protective effect of the antiplatelet medication aspirin in patients with COVID-19 may be related to the dose administered, an insensitivity to aspirin’s mechanism of platelet inhibition in COVID-19, or an altered platelet phenotype as was clearly demonstrated by Manne *et al.* comparing healthy platelets to platelets from patients with COVID-19^33^. Cameron *et al.* previously demonstrated a divergent platelet phenotype in patients with chronic arterial disease and diabetes with resistance to aspirin and clopidogrel therapy in diseased but not healthy platelets^21^. Similarly, Liang *et al* demonstrated in platelets from patients with diabetes, surface P2Y_12_ receptors are arranged in a different conformation and are impressively resistant to inhibition by clopidogrel ^35^.

Elbadawi *et al.* reported the absolute neutrophil count and not D-dimer, a traditional biomarker associated with thrombosis, is an independent predictor of thrombotic events in patients with COVID-19^36^. The mortality benefit of dexamethasone, an immunosuppressant and anti-inflammatory medication, in hospitalized patients with COVID-19^37^ and recent reports of immunothrombosis^17,38-41^ and microvascular occlusion^18,42-44^ by multiple independent groups suggest platelets may be indirect mediators of thrombosis and perhaps not the best direct targets for pharmacological intervention. Contemporaneous with submission of this manuscript, a smaller, non-propensity matched study has shown aspirin treatment decreased mortality that was driven by reduced ICU level care and mechanical ventilatory needs but not thrombosis in patients with COVID-19. This report suggests a protective effect of aspirin that is distinct from altering end-organ thrombosis ^45^, and possibly from immune-mediated acute respiratory distress syndrome (ARDS) as previously demonstrated^46,47^. By evaluating another anti-inflammatory mechanism using patients treated with NSAIDs in parallel with aspirin in the same hospital and locations in the U.S., we similarly show no effect on mortality, with all statistical models accounting for any contribution of prophylactic and therapeutic heparin use in hospitalized patients and subsequent outcomes.

The signal for increased composite thrombotic events in COVID-19 patients treated with aspirin was surprising and driven mostly by stroke. Recent observational studies show mixed results for COVID-19-related stroke risk with one small study suggesting an increased risk in younger patients^48^, one large study showing an overall low risk^49^, and one very large study paradoxically showing that COVID-19 infection is associated with a decreased risk of thrombotic cerebrovascular stroke^50^. A mechanistic explanation for our finding may be related to the known neuroprotective effect of interleukin-6 (IL-6)^51^ which is greatly elevated in systemic SARS-CoV-2 infection^52^ and reported to be reduced by aspirin^53^.

We show quite clearly in our study with investigators working independently of each other in different regions of the U.S. that human platelets contain the SARS-CoV-2 receptors ACE2 and TMPRSS2. The inter-individual expression difference of platelet ACE2 and TMPRSS2 was striking. Our overall observation is consistent with the findings of Zaid *et al.* who identified SARS-CoV-2 mRNA in human platelets implying a mechanism of entry must exist, and then a report by Zhang *et al.* who identified ACE2 on human platelets^20,54^. Our data are at odds with Manne *et al.* who failed to detect ACE2 protein in platelets by immunoblotting using white blood cells (WBC) as a positive control ^33^. Notably, Manne *et al.* employed a CD45 depletion step on isolated platelets to eliminate the possibility of WBC contamination prior to immunoblotting. CD45 is also present on platelets, and we previously demonstrated this step decreases the platelet yield available for immunoblotting ^22^. Lastly, Nassa *et al.* have very elegantly shown that the platelet transcriptome and proteome are dynamic and often mRNA to protein concordance is not observed but, rather, dependent on external platelet cues^55^. Overall, our data are congruent with Koupenova *et al.* suggesting that the ssRNA virus SARS-CoV-2 may behave similarly to the ssRNA influenza virus by utilizing platelets to modulate immune function that ultimately may lead to immunothrombosis^16^.

### Study Limitations

The observational nature of this study from just two hospitals has intrinsic limitations, and the small patient sample to allow for propensity matching limits generalizability of our findings. A few patients testing positive for SARS-CoV-2 were ambulatory and we relied on physician prescriptions making it impossible to confirm compliance to aspirin therapy.

## Conclusions

SARS-CoV-2 high affinity receptors are present in platelets from healthy individuals. This finding crucially suggests platelets may be involved in COVID-19 pathogenesis and the observed thrombotic phenotype. However, our real-world clinical data suggests regular intake of low dose aspirin does not protect against adverse thrombotic events or death in COVID-19 patients. Platelets are fastidious components of the circulatory system with a wide range of critical functions, including contributing to immunoinflammatory host responses. Thus, targeting platelet thrombotic function may alter its roles in other domains. The nuanced mechanisms of thrombosis in COVID-19 may be unique and deserves further investigation. The use of traditional antiplatelet agents may not protect against thrombotic events or mortality in COVID-19, but, in fact, cause harm. The awareness of this potential harm and role of randomized controlled drug trials in assessing the suitability of antiplatelet agents in COVID-19 is critical.

## Supplementary Material

Supplement

## Figures and Tables

**Table 1.** Characteristics of Population taking Aspirin Therapy: Unadjusted data are for patients testing positive for SARS-CoV-2 not taking aspirin or with established aspirin therapy or initiated with low dose aspirin at the time of diagnosis.

Factor	No Aspirin (N=1,709)		Aspirin Use (N=285)		p-value
	N	Statistics	N	Statistics	
***Medications***					
CLOPID	1,709	9 (0.53)	285	27 (9.5)	***<0.001***^c^
Ticag	1,709	1 (0.06)	285	6 (2.1)	***<0.001***^d^
Prasug	1,709	0 (0.00)	285	0 (0.00)	
Cangr	1,709	0 (0.00)	285	0 (0.00)	
Cilost	1,709	0 (0.00)	285	0 (0.00)	
Pentox	1,709	0 (0.00)	285	1 (0.35)	0.14^d^
AntiPlt	1,709	10 (0.59)	285	285 (100.0)	***<0.001***^c^
Multiple Therapy	1,709	0 (0.00)	285	34 (11.9)	***<0.001***^d^
AC_thcrputic	1,709	94 (5.5)	285	56 (19.6)	***<0.001***^c^
AC_prophylct	1,709	355 (20.8)	285	215 (75.4)	***<0.001***^c^
NSAIDs	1,650	294 (17.8)	260	171 (65.8)	***<0.001***^c^
***Covariates***					
Age	1,709	50.6 ± 17.5	285	70.0 ± 13.6	***<0.001***^a2^
Platelets	689	217.4 ± 79.3	253	208.7 ± 853	0.14^a1^
Gender	1,651		285		***<0.001***^c^
Male		804 (48.7)		172 (60.4)	
Female		847 (51.3)		113 (39.6)	
Race	1,564		280		***<0.001***^c^
White		948 (60.6)		144 (51.4)	
Black		506 (32.4)		124 (44.3)	
Other		110 (7.0)		12 (4.3)	
Ethnicity	1,480		277		***<0.001***^c^
Hispanic		204 (13.8)		7 (2.5)	
Non-Hispanic		1,276 (86.2)		270 (97.5)	
Smoking	1,417		268		***<0.001***^c^
No		924 (65.2)		123 (45.9)	
Former		362 (25.5)		124 (46.3)	
Current		131 (9.2)		21 (7.8)	
RespSuprt	1,709	191 (11.2)	285	117 (41.1)	***<0.001***^c^
OnPressors	1,709	81 (4.7)	285	47 (16.5)	***<0.001***^c^
HemodInstab	1,709	85 (5.0)	285	48 (16.8)	***<0.001***^c^
COPD_cmphysema	1,399	82 (5.9)	274	53 (19.3)	***<0.001***^c^
Asthma	1,410	243 (17.2)	273	66 (24.2)	***0.007***^c^
Diabetes	1,424	318 (22.3)	278	147 (52.9)	***<0.001***^c^
Hypertension	1,447	659 (45.5)	281	244 (86.8)	***<0.001***^c^
Coronary _artery_disease	1,405	116 (8.3)	275	100 (36.4)	***<0.001***^c^
Heart_Failure	1,404	108 (7.7)	274	78 (28.5)	***<0.001***^c^
Cancer	1,447	184 (12.7)	280	63 (22.5)	***<0.001***^c^
On_immunosuppressive_treatment	1,456	144 (9.9)	277	36 (13.0)	0.12^c^

Statistics presented as Mean ± SD, N (column %). p-values:

a1= t test

a2= Satterthwaite t-test

c= Pearson's chi square test

d= Fisher's Exact test.

**Table 2.** Characteristics of Population taking NSAID Therapy: Unadjusted data are for patients testing positive for SARS-CoV-2 not taking NSAID or with established NSAID therapy or initiated with NSAID at the time of diagnosis.

Factor	No NSAIDs (N=1,445)		NSAIDs (N=465)		p-value
	N	Statistics	N	Statistics	
***Medications***					
CLOPID	1,445	12 (0.83)	465	21 (4.5)	***<0.001***^c^
Ticag	1,445	0 (0.00)	465	7 (1.5)	***<0.001***^d^
Prasug	1,445	0 (0.00)	465	0 (0.00)	
Cangr	1,445	0 (0.00)	465	0 (0.00)	
Cilost	1,445	0 (0.00)	465	0 (0.00)	
Pentox	1,445	0 (0.00)	465	1 (0.22)	0.24^d^
AntiPlt	1,445	96 (6.6)	465	174 (37.4)	***<0.001***^c^
Multiple Therapy	1,445	5 (0.35)	465	26 (5.6)	***<0.001***^c^
AC_therputic	1,445	95 (6.6)	465	45 (9.7)	***0.026***^c^
AC_prophylct	1,445	328 (22.7)	465	203 (43.7)	***<0.001***^c^
***Covariates***					
Age	1,445	51.5 ± 18.2	465	58.9 ± 17.1	***<0.001***^a^
Platelets	574	213.1 ± 80.8	314	212.8 ± 78.0	0.97^a^
Gender	1,390		462		0.36^c^
Male		688 (49.5)		240 (51.9)	
Female		702 (50.5)		222 (48.1)	
Race	1,310		451		***0.005***^c^
White		801 (61.1)		243 (53.9)	
Black		416 (31.8)		181 (40.1)	
Other		93 (7.1)		27 (6.0)	
Ethnicity	1,228		454		***<0.001***^c^
Hispanic		178 (14.5)		31 (6.8)	
Non-Hispanic		1,050 (85.5)		423 (93.2)	
Smoking	1,162		452		***<0.001***^c^
No		763 (65.7)		247 (54.6)	
Former		303 (26.1)		160 (35.4)	
Current		96 (8.3)		45 (10.0)	
RespSuprt	1,445	200 (13.8)	465	85 (18.3)	***0.019***^c^
OnPressors	1,445	91 (6.3)	465	27 (5.8)	0.70^c^
HemodInstab	1,445	94 (6.5)	465	29 (6.2)	0.84^c^
COPD_emphysema	1,161	74 (6.4)	440	54 (12.3)	***<0.001***^c^
Asthma	1,168	199 (17.0)	442	100 (22.6)	***0.010***^c^
Diabetes	1,179	278 (23.6)	450	164 (36.4)	***<0.001***^c^
Hypertension	1,198	571 (47.7)	453	292 (64.5)	***<0.001***^c^
Coronary_artery_disease	1,162	107 (9.2)	445	98 (22.0)	***<0.001***^c^
Heart_Failure	1,162	105 (9.0)	444	70 (15.8)	***<0.001***^c^
Cancer	1,202	164 (13.6)	447	75 (16.8)	0.11^c^
On_immunosuppressive_treatment	1,209	119 (9.8)	446	55 (12.3)	0.14^c^
History _of_transplant	1,159	9 (0.78)	443	10 (2.3)	***0.014***^c^

Statistics presented as Mean ± SD, N (column %).

p-values:

a= t-test

c= Pearson's chi-square test

d= Fisher's Exact test.
