# Supplementary material for: SARS-CoV-2 Receptors are Expressed on Human Platelets and the Effect of Aspirin on Clinical Outcomes in COVID-19 Patients
Source: Res Sq. 2020 Dec 23:rs.3.rs-119031. Preprint. [Version 1] doi: 10.21203/rs.3.rs-119031/v1 (PMC7781327; doi:10.21203/rs.3.rs-119031/v1)
Supplement: Supplement [file 8d2f580692c1159653da96a6.pdf]

SARS-CoV-2 Receptors are Expressed on Human Platelets and the Effect of Aspirin on Clinical Outcomes in COVID-19 Patients

Aditya Sahai MD [1‡], Rohan Bhandari MD[1,2‡], Milka Koupenova PhD[3], Jane E. Freedman MD[3], Matthew Godwin BS[1], Thomas McIntyre PhD[2,4], Mina K. Chung MD[1,2,4], Jean-Pierre Iskandar MD[5], Hayaan Kamran MD[1], Essa Hariri MD[5], Anu Aggarwal PhD[2], Ankur Kalra MD[1], John R. Bartholomew MD[1,4], Keith R. McCrae MD[4,6], Ayman Elbadawi MD[7], Lars G. Svensson MD PhD[1,4], Samir Kapadia MD[1,4] and Scott J. Cameron MD PhD[1,2,4 *]

Running Title: Platelets Express SARS-CoV-2 Receptors & Aspirin Does Not Protect in COVID-19

Author Affiliations:

Heart Vascular and Thoracic Institute, Cleveland Clinic, Cleveland, OH
Department of Cardiovascular and Metabolic Sciences, Cleveland Clinic, Cleveland, OH
Department of Medicine, University of Massachusetts Medical School, Worcester, MA.
Case Western Reserve University Cleveland Clinic Lerner College of Medicine, Cleveland, OH
Department of Medicine, Cleveland Clinic, Cleveland, OH
Taussig Cancer Institute, Cleveland Clinic, Cleveland, OH
Division of Cardiovascular Medicine, University of Texas Medical Branch, Galveston, TX

[‡]Equally contributing authors

Word Count: 2995 (main text), 199 (abstract)
References: 55
Figures: 4
Tables: 2

Address for Correspondence*: Cleveland Clinic Foundation, Department of Cardiovascular Medicine, Section of Vascular Medicine, J3-5, 9500 Euclid Avenue, Cleveland, OH, 44195, USA. Telephone: (216) 316-6794. Email: cameros3@ccf.org

Key Words: Platelets, SARS-CoV-2, COVID-19, Thrombosis, ACE2, TMPRSS2

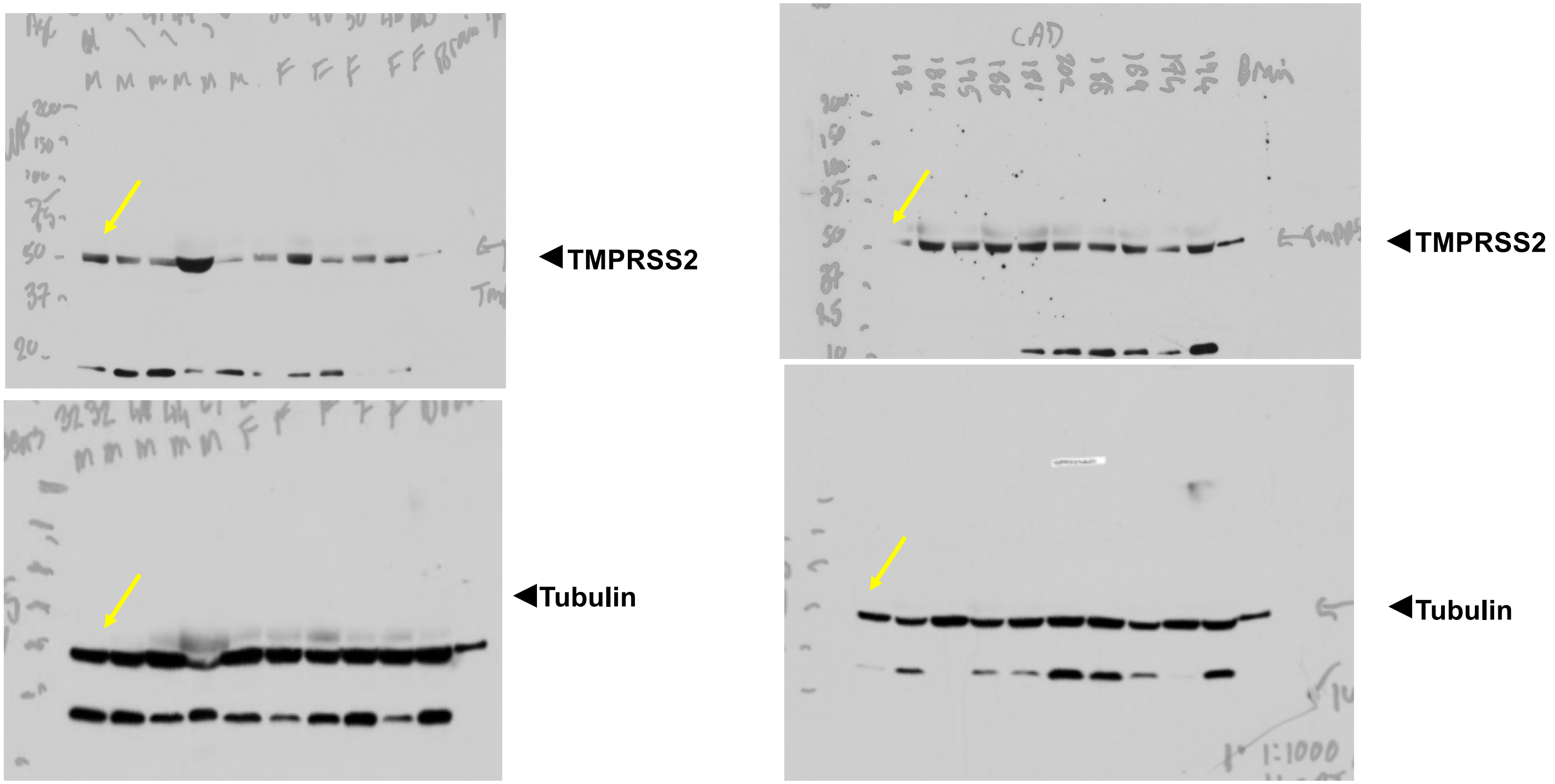

Supplemental Fig 1 (A). Expression of TMPRSS2 in Platelets: Washed platelets from healthy individuals (mean age 40.1 ± 2.8 years, n=20) were isolated and proteins separate by SDS-PAGE with molecular weight shown in KiloDaltons (KDa). Immunoblotting was conducted an using an anti-TMPRSS2 antibody or anti-tubulin immunoblotting as a loading control. The ratio of protein to loading control is expressed as a function of age and the correlation coefficient is noted (r ± 95% CI, P=0.30). Human brain lysate served as a positive control for TMPRSS2 migrating at the expected molecular weight (~50 KDa). For clarity of presentation, the tubulin blot was cropped just above and below the 50 KDa marker line. A yellow arrowhead indicates the position of the immunoreactive band.

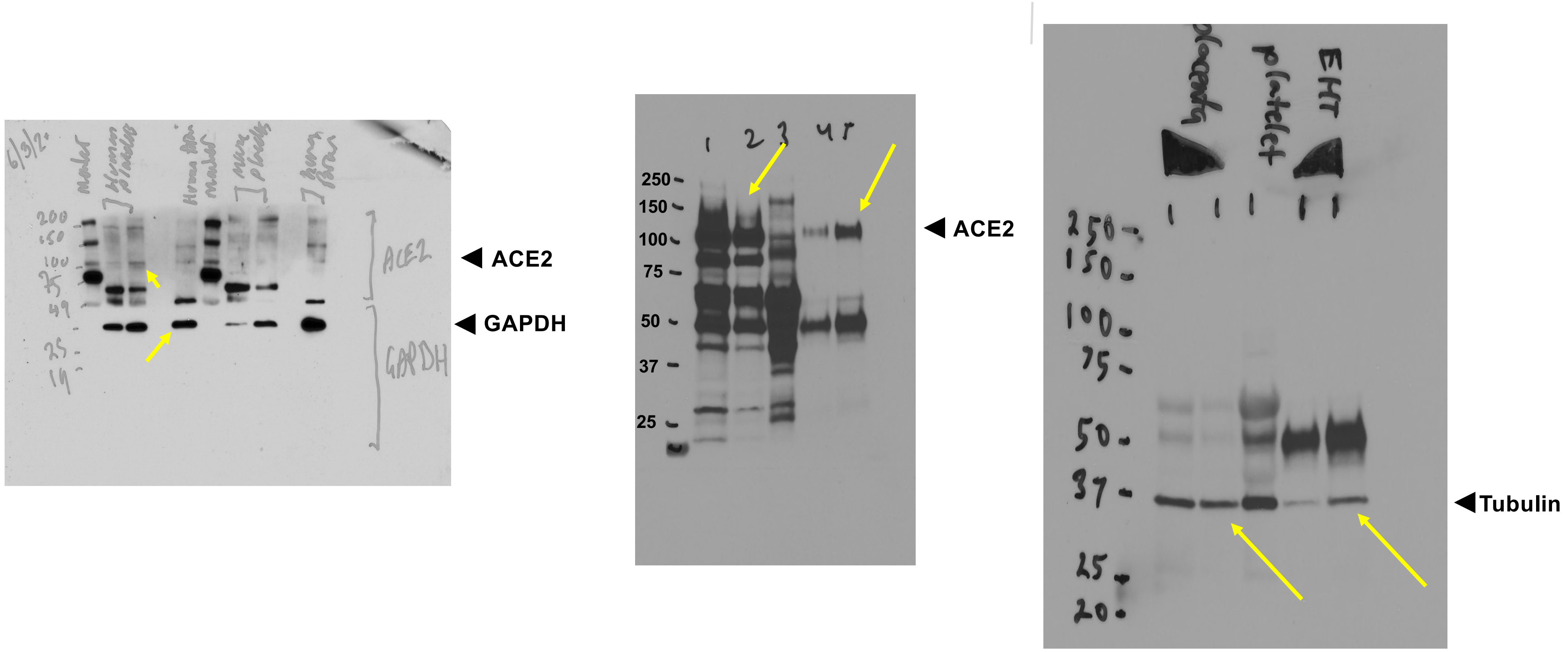

Supplemental Fig 1 (B). Expression of ACE2 in Platelets: Washed platelets from healthy individuals (mean age 40.1 ± 2.8 years, n=20) were isolated and proteins separate by SDS-PAGE with molecular weight shown in KiloDaltons (KDa). Lane 1 is human platelet lysate, lane 2 is human brain lysate, lane 3 is human placenta lysate, lane 4 is lysate from engineered human heart tissue. Immunoblotting was conducted using an using anti-ACE2 antibody. Anti-tubulin and anti-GAPDH are loading controls. ACE2 migrates at the expected molecular weight (~100 KDa) shown by an arrowhead with glycosylated forms indicated by *. For clarity of presentation, the tubulin and GAPDH blots are cropped just above and below the 50 Kda and 36 KDa marker lines, respectively and the ACE2 blot is cropped just below the 75 KDa marker. The grey partition line for ACE2 and tubulin are from the same blot separated by three lanes. The yellow arrow denoted the position of the immunoreactive band.

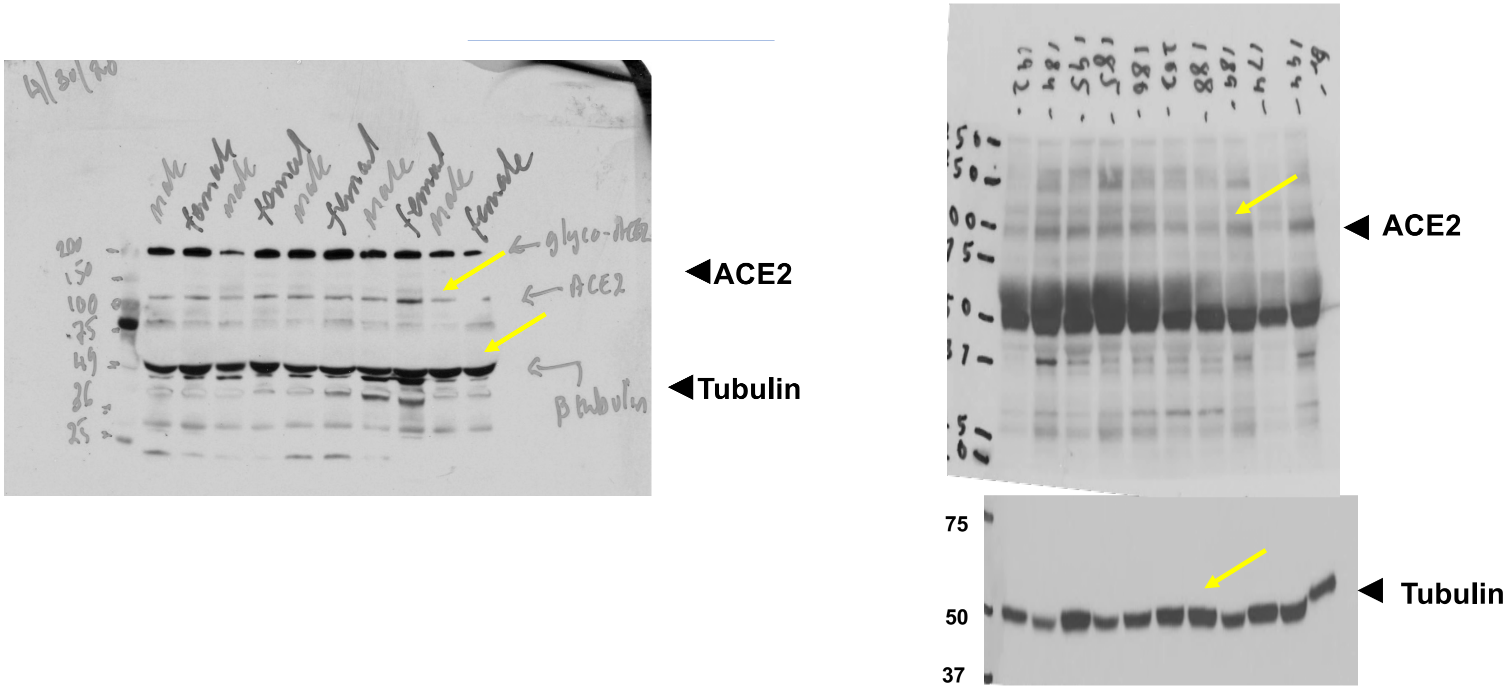

Supplemental Fig 1 (C). Expression of ACE2 in Platelets: Washed platelets from healthy individuals (mean age 40.1 ± 2.8 years, n=20) were isolated and proteins separate by SDS-PAGE with molecular weight shown in KiloDaltons (KDa). Lane 1 is human platelet lysate, lane 2 is human brain lysate, lane 3 is human placenta lysate, lane 4 is lysate from engineered human heart tissue. Immunoblotting was conducted using an using anti-ACE2 antibody. Anti-tubulin and anti-GAPDH are loading controls. ACE2 migrates at the expected molecular weight (~100 KDa) shown by an arrowhead with glycosylated forms indicated by *. For clarity of presentation, the tubulin and GAPDH blots are cropped just above and below the 50 Kda and 36 KDa marker lines, respectively and the ACE2 blot is cropped just below the 75 KDa marker. The ACE2 nitrocellulaose membrane on the left was divided in two just below the 75 KDa marker line, with the top half probed with ACE2 antibody and the bottom half probed with tubulin antibody. The yellow arrow denoted the position of the immunoreactive band.

	No Aspirin (N=248)	Aspirin (N=248	p-value
Death	38 (15.3)	33 (13.3)	0.52
Thrombotic Stroke	1 (0.40)	9 (3.6)	***0.036***
MI	2 (0.81)	5 (2.0)	0.27
VTE	4 (1.6)	10 (4.0)	0.12
Composite Endpoint (MI, Thrombotic Stroke, VTE)	7 (2.8)	23 (9.3)	***0.005***

Statistics presented as N (column %).

	No NSAIDs (N=444)	NSAIDs (N=444)	p-values
Death	32 (7.2)	31 (7.0)	0.90
Thrombotic Stroke	2 (0.45)	5 (1.1)	0.27
MI	1 (0.23)	3 (0.68)	0.34
VTE	4 (0.90)	9 (2.0)	0.17
Composite Endpoint (MI, Thrombotic Stroke, VTE)	7 (1.6)	17 (3.8)	***0.046***

Statistics presented as N (column %).

Supplemental Fig 2. Summarized Primary and Secondary Outcomes: Propensity-matched data for patients testing positive for SARS-CoV-2 and outcomes taking either 81 mg aspirin (n=248 in each group) or NSAID (n=444 in each group) at the time of diagnosis. The composite endpoint is: MI, thrombotic stroke, VTE.
